# Complete genome sequence of *Erythrobacteraceae* bacterium WH01K, a strain isolated from corals (*Acropora* sp.) in Taiwan

**DOI:** 10.1128/MRA.00830-23

**Published:** 2023-11-07

**Authors:** Shih-Hsun Walter Hung, I-Chen Wu, Chieh-Chen Huang, Chih-Horng Kuo

**Affiliations:** 1 Department of Life Sciences, National Chung Hsing University, Taichung, Taiwan; 2 Institute of Plant and Microbial Biology, Academia Sinica, Taipei, Taiwan; 3 Advanced Plant and Food Crop Biotechnology Center, National Chung Hsing University, Taichung, Taiwan; 4 Innovation and Development Center of Sustainable Agriculture, National Chung Hsing University, Taichung, Taiwan; 5 Biotechnology Center, National Chung Hsing University, Taichung, Taiwan; University of Southern California, Los Angeles, California, USA

**Keywords:** genome, *Erythrobacteraceae*, symbiont, coral, *Acropora*

## Abstract

Here, we report the isolation and genome sequence of a coral-associated *Erythrobacteraceae* bacterium, strain WH01K. The complete assembly consists of one 2,745,896 bp circular chromosome and one 172,502 bp circular plasmid.

## ANNOUNCEMENT

Various bacteria associated with corals are known to benefit their hosts (1, 2) and are promising sources for biostimulants. To examine these resources, samples of corals (*Acropora* sp.) from Liuqiu Island (Pingtung, Taiwan; 22.346363 N 120.389678 E) were collected by scuba diving into 5 m depth on 2 May 2022. The samples were homogenized and streaked on marine agar (212185; BD Difco, USA) several times to obtain pure isolates. One strain WH01K with red-orange-color colonies was selected for this study. The procedures for genome sequencing and assembly were based on those described in our previous work (3). All kits were used according to the manufacturer’s instructions, and all bioinformatics tools were used with the default settings unless stated otherwise.

For DNA extraction, a single colony was picked and grown in marine broth (279110; BD Difco, USA) at 30°C for 48 h. The same inoculum was used for both sequencing platforms. For Illumina sequencing, total DNA was extracted using Wizard Genomic DNA Purification Kit (A1120; Promega, USA), followed by library preparation using KAPA LTP Library Preparation Kit (07961880001; Roche, Switzerland). The Illumina NovaSeq 6000 2 × 150 bp paired-end sequencing (S4 Reagent Kit v1.5) produced 12,079,554 reads totaling 1.8 Gb. For Oxford Nanopore Technologies (ONT) sequencing, Nanobind CBB Kit (102-301-900; PacBio, USA) was used for DNA extraction. The ONT Ligation Sequencing Kit (SQK-LSK114) was used without shearing for library construction and then sequenced on a MinION with a R10.4.1 flow cell (FLO-MIN114). Basecalling was performed using Guppy v6.4.2 with the high accuracy mode. A total of 6,097 raw reads were generated (combined length = 111.3 Mb, N50 = 29,385 bp). The hybrid *de novo* assembly was performed using Unicycler v0.4.9b (4) without pre-processing the reads. For validation, the Illumina and ONT raw reads were mapped to the assembly using BWA v0.7.17 (5) and Minimap2 v2.15 (6), respectively. The mapping results were programmatically checked using SAMtools v1.9 (7) and manually inspected using IGV v2.11.1 (8); no error was found.

The assembly consists of one chromosome (2,745,896 bp; 63.5% G+C) and one plasmid pWH01K (172,502 bp; 59.5% G+C; encodes one *repA*). The Illumina and ONT reads provided 625- and 38-fold coverage, respectively. The annotation by NCBI Prokaryotic Genome Annotation Pipeline (PGAP) v6.4 (9) predicted 3 rRNA genes, 43 tRNA genes, 4 non-coding RNAs, and 2,817 protein-coding genes. Based on average amino acid identity (AAI) analysis using Enveomics v2022.12.15 (10), this strain has ~64-70% AAI with representative members of *Erythrobacteraceae* such as *Qipengyuania*, *Pseudopontixanthobacter*, and *Aurantiacibacter*. The low levels of genome similarity prevented confident classification at the genus level for this bacterium. The benchmarking universal single-copy ortholog (BUSCO) analysis v5.1.2 (11) with the *Sphingomonadales* data set identified 1,011 complete and single-copy BUSCOs (99.3%), two fragmented BUSCOs (0.2%), and five missing BUSCOs (0.5%). The assessment, together with the finding that both the chromosome and plasmid are represented by circular contigs, suggested that this genome assembly is complete.

## Data Availability

This genome project has been deposited in NCBI under the accession number PRJNA922479. The Illumina and ONT raw reads have been deposited at the NCBI Sequence Read Archive under the accession numbers SRR23047363 and SRR23047364, respectively. The genome sequence has been deposited in GenBank under the accession numbers CP115966 (chromosome) and CP115967 (plasmid).
